# An update to the custom-made MS Excel workbook performing the log-rank test with extended functionality and a new original COVID-19 training data set

**DOI:** 10.3325/cmj.2021.62.531

**Published:** 2021-10

**Authors:** Marko Lucijanić

**Affiliations:** 1 Hematology Department, Dubrava University Hospital, Zagreb, Croatia; 2 Primary Respiratory and Intensive Care Center, Dubrava University Hospital, Zagreb, Croatia; 3 University of Zagreb, School of Medicine, Zagreb, Croatia *markolucijanic@yahoo.com*

Free but functional statistical software is highly needed but hard to find. The unavailability of statistical software able to perform, and thus practice and learn, survival analysis is one of the main reasons why this area of statistics remains unnecessary mystified and unreachable to the medical professionals who need it the most – young researchers at the start of their careers with limited financial and institutional resources. Several custom-made MS Excel workbooks performing time-to-event analyses have been developed in the recent years by the author of this paper (1-3). They cover specific topics as seen from my point of view and represent a personal learning curve on survival analysis. A straightforward statistical program intended for quick data grinding and screening for possible survival associations was published in the *Croatian Medical Journal* in 2016 (2). It was deliberately limited to a maximum of 300 cases to avoid long calculation times of MS Excel. The current updates have been created as a response to a number of requests to extend the workbook to deal with larger data sets. Additional functionality and new training data sets have also been added (described below).

Three updated versions of the workbook can be downloaded from the links provided in this article. The workbooks have been prepared for 300 cases (Supplementary file 1)(Supplementary file 1), 600 cases (Supplementary file 2)(Supplementary file 2), and 1200 cases (Supplementary file 3)(Supplementary file 3). They increase in size and calculations become more cumbersome with an increasing number of rows. The workbook created for the lowest number of cases that will fit your needs should be used.

The new training data sets included with the updated program versions consist of real-life data from the Dubrava University Hospital registry of hospitalized COVID-19 patients. Six-month overall survival for patients with acute COVID-19 infection can be assessed by choosing the “OS 6mth” variable as time and “status 6mth” variable as the censoring status. The group variable can be chosen from a long list of COVID-19-related parameters, comorbidities, drugs in chronic therapy, or admission laboratory data.

**Figure 1:**
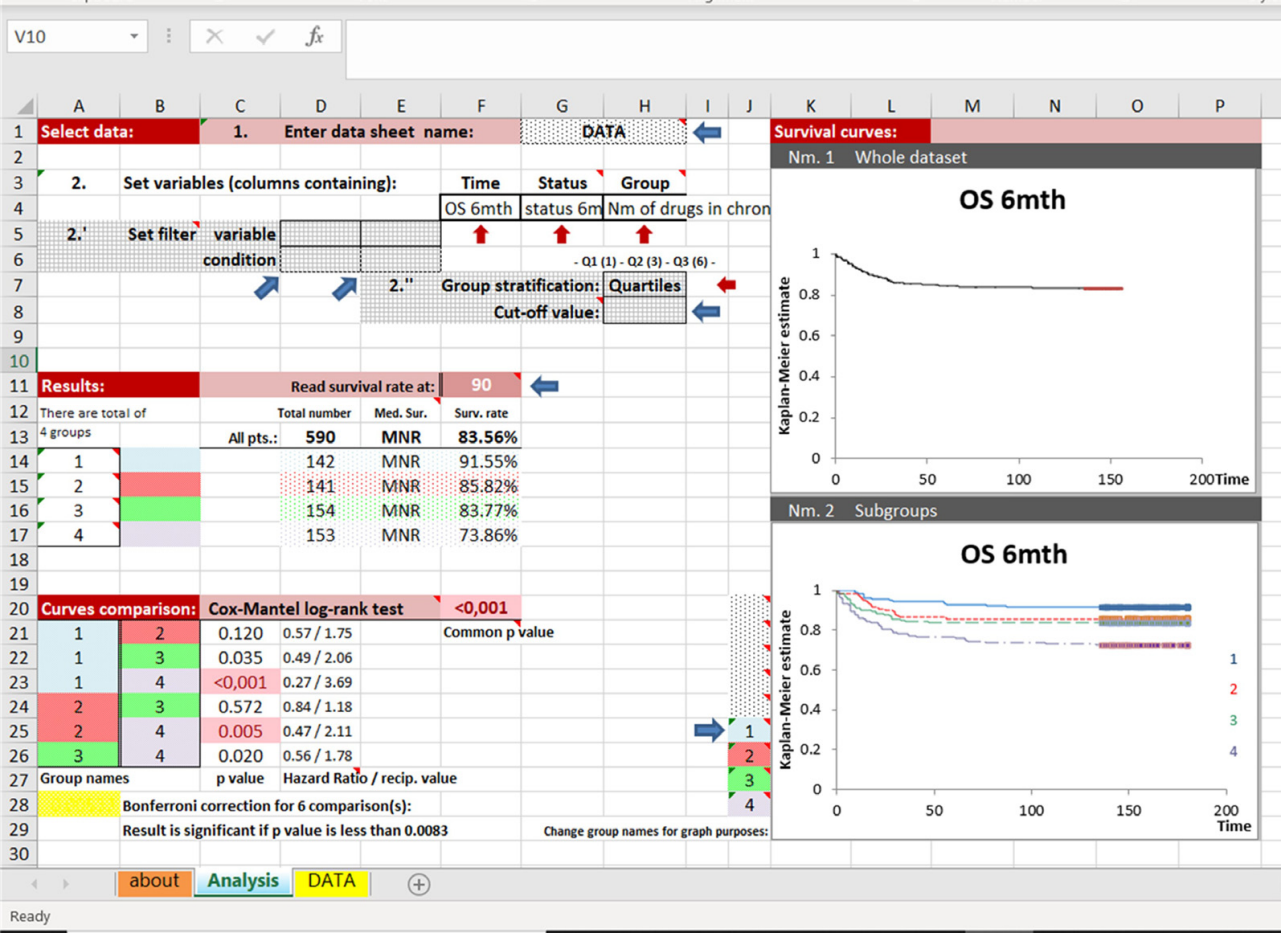
User interface placed on the “Analysis” sheet of all three workbooks. The workbook for 600 cases is shown.

Updated versions have kept the user interface created for the original program version. The new interface is shown in Figure 1. Users are able to sequentially choose which data will be analyzed. In the first step (1.) users choose the name of the sheet that contains data (marked with a blue arrow; set to “DATA” sheet where training data set is placed, but a new sheet with your data can be copied into the workbook). In the second step (2.) they can choose variables (columns) from the drop-down menus by their names and determine which variables will be used for the time and censoring status and which for subgroup comparisons (marked with red arrows). In the updated version, 256 columns of data placed on the “DATA” sheet can be loaded into the drop-down menus (up to “IV” column on the “DATA” sheet). In the subsequent steps (2.’), filters can be applied to the data based on two different variables and their conditions (marked with inclined blue arrows) and (2.”) users can choose to optionally stratify the group variable (marked with a red arrow). This new feature enables the use of numerical variables for subgroup comparisons. Group stratification is set to “Quartiles” by default, but numerical variables can be stratified by median, terciles, quartiles, or a custom cut-off value, or “None” option can be chosen. The median, terciles, and quartiles will be created if this option is chosen from the drop-down menu and if the group variable of interest has more than four unique values (more than four levels of data). They will not be created if the variable has only two levels, such as sex (male and female), even if chosen. When the option for the median, terciles, or quartiles is chosen, the program will show the median value, 33rd, and 66th percentile (termed T1 and T2) or 25th, 50th, and 75th percentile (termed Q1, Q2, and Q3), respectively. The patients belonging to the percentile of interest are included in the higher group (eg, patients belonging to Q3 are included in the highest quartile). The cut-off option (marked by a blue arrow) can be used with a group variable with less than four levels as it overrides the formula created for median/terciles/quartiles. This option enables users to dichotomize their data using a criterion they are interested in and investigate the relationship between the newly formed subgroups. As in the previous program version, the obtained results are summarized below the input columns. The time of interest when the user wishes to assess the time-to-event rate can be additionally tweaked to obtain the needed estimates (marked with a blue arrow; set to 90 by default in this update, which corresponds to a three-month survival rate since survival in training data sets is measured in days). The curves are compared between the subgroups using the Cox-Mantel version of the log-rank test. *P* values and hazard ratios are presented. An overall time-to-event curve and curves stratified by the subgroups of interest are presented in two figures as in the original version. The results can be printed out on a single A4 page in landscape orientation.

I would like to briefly comment on the results presented in the second figure in Figure 1 (and corresponding analyses in different versions of the updated workbook). These curves represent the association of a higher number of drugs in chronic therapy and a worse six-month survival of acute COVID-19 patients. As you can observe if analyzing the same issue in different supplementary workbooks (with different sample sizes), statistical significance for particular comparisons between the curves is more likely to be obtained if using larger data sets, although the curves for subgroups of patients have overall similar appearance. If we only observe the appearance of the curves and not statistical significance of mutual comparisons, the steepest drop in the curves is present at the start of the follow-up, ie, during acute COVID-19 infection, but deaths continue to occur up to about three months of follow-up, when the plateau is reached in patients belonging to lower quartiles. In the 300-row workbook, no patients belonged to the lowest quartile as Q1 was 0, which was the lowest possible value recorded. These patients were automatically included in the higher quartile as explained above, and only three curves are presented. It is very important to state that, since these were retrospectively collected data, no causal relationship can be inferred from these results (ie, that polypharmacy causes more COVID-19 deaths) and only associations can be assessed (ie, patients with higher number of drugs in chronic therapy are more likely to die from COVID-19). Furthermore, censored observations are grouped at the ends of survival curves (markers on the curves), which is not a software error but a representation of the fact that patients in training datasets were followed up for a substantial period of time.

In conclusion, new workbook versions have been tested on several different data sets and seemed to have worked as expected during the testing period. One should bear in mind that any software can have bugs, which might become evident with the future use. If you find any, please contact me to update the software. Use these workbooks with caution and periodically compare your results with those obtained by commercial statistical packages. I hope that these tools will help you learn and use survival analysis in your everyday clinical practice, and ultimately help you publish your real-life observations.
